# Scaffold analysis of PubChem database as background for hierarchical scaffold-based visualization

**DOI:** 10.1186/s13321-016-0186-7

**Published:** 2016-12-29

**Authors:** Jakub Velkoborsky, David Hoksza

**Affiliations:** Faculty of Mathematics and Physics, Charles University, Prague, Czech Republic

**Keywords:** Visualization, Scaffold, Treemap, Pubchem, Chemical space

## Abstract

**Background:**

Visualization of large molecular datasets is a challenging yet important topic utilised in diverse fields of chemistry ranging from material engineering to drug design. Especially in drug design, modern methods of high-throughput screening generate large amounts of molecular data that call for methods enabling their analysis. One such method is classification of compounds based on their molecular scaffolds, a concept widely used by medicinal chemists to group molecules of similar properties. This classification can then be utilized for intuitive visualization of compounds.

**Results:**

In this paper, we propose a scaffold hierarchy as a result of large-scale analysis of the PubChem Compound database. The analysis not only provided insights into scaffold diversity of the PubChem Compound database, but also enables scaffold-based hierarchical visualization of user compound data sets on the background of empirical chemical space, as defined by the PubChem data, or on the background of any other user-defined data set. The visualization is performed by a web based client-server application called Scaffvis. It provides an interactive zoomable tree map visualization of data sets up to hundreds of thousands molecules. Scaffvis is free to use and its source codes have been published under an open source license.

**Graphical abstract:**
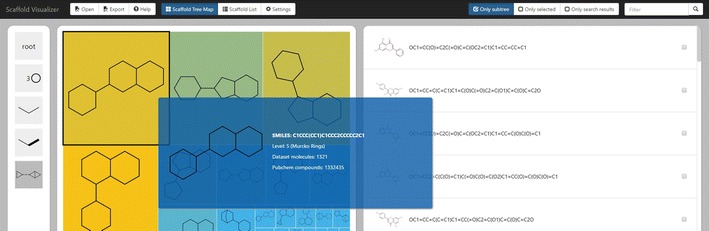
.

**Electronic supplementary material:**

The online version of this article (doi:10.1186/s13321-016-0186-7) contains supplementary material, which is available to authorized users.

## Background

With the growing sizes of existing chemical libraries it is becoming increasingly important to be able to explore and analyze those libraries to gain insight into the their composition. For this purpose, visualization is an indispensable tool. It helps users to inspect types of compounds present in the library or understand how the compounds are organized. This is especially true when dealing with heterogeneous libraries.

When visually exploring a set of molecules, two distinct approaches can be taken. First, the molecules can be depicted individually, embedded into a plane or a three-dimensional space, based on selected properties and employing techniques of dimensionality reduction or multidimensional scaling. The other approach is to define a hierarchical structure over the molecules which allows the molecules to be visualized in groups at different levels of the hierarchy.

In the direct visualization approach, molecules are drawn as individual entities. There is no universal way to map molecules to coordinates in an Euclidean space (of preferably at most three dimensions) and it is nontrivial to devise a reasonable and practically useful mapping. Starting with a set of molecules to be visualized, the process of calculating their coordinates is usually two-phase—first a property space is created which is then projected to a two- or three-dimensional Euclidean space. The property space is obtained by choosing a set of molecular properties that is supposed to drive the visualization. From these properties we either get coordinates in a high dimensional Euclidean space (e.g. choosing numerical properties and using the properties’ values as the coordinates) or define a metric based on similarity of the molecules (e.g. molecular fingerprints and Tanimoto distance) yielding a metric space. Once a property space is defined, a suitable projection method is used to calculate the desired coordinates. The most common projection techniques include principal component analysis [1–3], multidimensional scaling [4], generative topographic mapping [5, 6] or force-directed graph drawing [7]. There are two main disadvantages to direct visualization. First, visualizing large datasets with tens of thousands of compounds might be sometimes impractical due to screen size limitations; second, mapping molecules to coordinates is often itself dependent on the original input. That means that the same molecule pictured in two different datasets might be assigned different coordinates. It also means that if we add new molecules to a dataset, the position of the original molecules might change. However, this latter problem can be targeted by creating more global data set independent mappings, such as the ChemGPS [1] and MQN (Molecular Quantum Numbers) [2].

The other approach to visualization of molecular datasets is hierarchical visualization. One way of obtaining a suitable hierarchy is hierarchical clustering where one can employ, again, the molecular fingerprint-based similarity and use it to cluster the compounds with an arbitrary hierarchical clustering algorithm. Another way is to create a hierarchy based on structural features of the molecules, i.e. molecules sharing common structural features on given level are clustered together. A natural way is to use a hierarchy based on molecular scaffolds, a concept widely used in medicinal chemistry [8]. Generally, molecular scaffold represents the core of a molecule without its functional groups. Such description is general enough to allow for different scaffold definitions. One approach can define molecular scaffold as the original molecule with its R-groups removed. A more general approach can, in addition, transform all heteroatoms to carbons or even shorten the linkers between rings to minimum lengths. In this way, one can build a hierarchy of scaffolds allowing to see a molecule on different levels of abstraction. We call such hierarchy a scaffold hierarchy. Existing scaffold hierarchies eligible for the hierarchical visualization task include, for example, HierS [9], Scaffold Tree [10], Scaffold topologies [11] or the scaffold hierarchy introduced in this work. There even exist few approaches implementing hierarchical scaffold-based visualization such as Scaffold Hunter [12] or Scaffold Explorer [13]. While Scaffold Hunter provides visualization based on the Scaffold Tree (as well as on hierarchical clustering), the scaffolds in Scaffold Explorer are defined interactively by the user at the time of performing the interactive analysis.

In this paper, we introduce a novel approach capable of hierarchical scaffold-based visualization of chemical libraries on the background of empirical chemical space as defined by human chemists. The approach is implemented as a web-based tool called Scaffvis, allowing to explore molecular scaffolds in the form of a zoomable tree map. Unlike existing tools, it does so on the scaffold background of known chemical space defined by the PubChem database [14]. The relative frequency of a user scaffold in the background hierarchy is by default encoded by the size of the treemap squares while their color encodes the frequency of a scaffold in the user data set.

## Methods

To be able to visualize a molecular dataset with different levels of detail with respect to the molecular scaffolds, we need a set of scaffold definitions forming a hierarchy, optimally a tree hierarchy. That is if two compounds share a scaffold at given level, they also share scaffold at all the levels above. In the following sections we introduced existing hierarchies, discuss their pros and cons and then propose our extended scaffold hierarchy.

### Existing scaffold hierarchies

When looking for possible hierarchies for our purpose, we considered three existing scaffold hierarchies, namely HierS [9], Scaffold Tree [15] and Scaffold topologies [11].

HierS method starts from a molecular framework (called a superscaffold in that context), takes one by one each of its ring systems (i.e. cycles sharing an edge), removes it (together with corresponding linkers, i.e. atoms connecting rings) and continues recursively on the rest of the molecule. This yields scaffolds being all possible ring system combinations of the original framework. Disadvantage of such method is that for each molecular framework with more than one ring system we get multiple scaffolds and for a framework with one ring system only we get the framework itself. Thus no obvious tree hierarchy is formed. The authors of the HierS algorithm order all the scaffolds (generated from a set of molecules/framework) by inclusion and form a hierarchy from this set with the minimal scaffolds being at the top and each next level having one additional ring system. Unfortunately, such hierarchy is not a tree (nor a forest). Also worth mentioning is that the algorithm never breaks fused rings in a complex ring system.

Scaffold Tree, proposed by Schuffenhauer at al [10, 15], uses a similar basic idea of iterative ring removal, but applies it in a very different way. A less important modification is that the algorithm only removes one ring at a time, not the whole ring system. But the key difference is that the rings are removed deterministically by a list of priorities and the algorithm never backtracks. For each molecular framework, we get a sequence of scaffolds linearly ordered by inclusion—each following scaffold having one less ring and being contained in the previous one. Such scaffolds are sometimes referred to as Schuffenhauer scaffolds. The clear advantage over HierS is that scaffolds obtained in this way form a tree hierarchy. The disadvantage is that a ring system that is more important for the molecule’s function might be removed first and the molecule might be on higher levels represented by a ring system which is not functionally relevant.

In [11], the concept of scaffold topology was introduced, which abstracts on the idea of a graph framework introduced by Bemis and Murcko [16]. There, the molecule is decomposed into a framework (often referred to as Murcko or molecular framework) and side chains. Framework is defined as a union of ring systems and linker atoms connecting the ring systems together. Side chains are the rest of the molecule—non-ring, non-linker atoms. [16] also defines graph frameworks, which only consider (heavy) atom connectivity and disregard atom type, hybridization and bond order. These graph frameworks are sometimes called the Murcko scaffolds. Scaffold topology is then defined as a “connected graph with the minimal number of nodes and corresponding edges required to fully describe its ring structure”. We can obtain such topology from a graph framework by iterative replacement of vertices of degree two by a single edge—i.e. the procedure of edge merging, a process inverse to edge subdivision.^1^ Such topologies are often referred to as Oprea scaffolds. There is one unique Oprea scaffold for each molecule. Oprea scaffold itself is actually not a hierarchy, but together with molecular frameworks and Murcko scaffolds, the Oprea scaffolds form a clearly defined tree hierarchy corresponding to topological configuration of ring systems in a molecule with different degree of abstraction at each level. This corresponds to how medicinal chemists intuitively perceive molecules.

### Proposed scaffold hierarchy

There are conditions which a scaffold hierarchy should fulfill to be used for understandable visualization; the hierarchy should have a tree topology and branching factor of the tree nodes should be homogenous.

The advantage of using a tree hierarchy is that each molecule can be classified by a unique representative at every level, as was already mentioned; but also that the hierarchy is largely independent of the molecules—knowing a given level scaffold we can compute scaffolds on all higher levels without the original molecule. That also implies that the hierarchy can be largely precomputed, using a sufficiently large set of representative and diverse molecules.

For the purpose of visualization, it would be ideal if the tree had a homogeneous branching factor—i.e. if all nodes had a similar number of children. Even when we do not reach homogenous branching factor, the number of children for each node should be limited to a reasonable number which can be laid out on the screen. Since the introduced scaffold hierarchies do not fulfill this criterion (see the next section for detailed analysis), we had to come up with a new hierarchy which achieves better branching factor on each level on the dataset corresponding to the empirical chemical space, i.e. on all molecules in PubChem. The hierarchy built from this set then serves as the background hierarchy for our visualization.

Our proposed scaffold hierarchy forms a rooted tree with a single virtual hierarchy root (level 0), eight levels of scaffolds (numbered 1–8), and molecules as leaves (level 9). That means that each molecule is mapped to a sequence of exactly eight scaffolds, one for each level. Each molecule and scaffold also has one uniquely defined parent. 9 levels is enough to cover the entire existing chemical space with a branching factor 100. We used PubChem Compound as the reference chemical space, so the we needed to cover about $10^8$ molecules (leaves). A tree of height 9 and constant branching factor 100 has $100^9 = 10^{18}$ leaves, which is indeed sufficient. In fact, for a tree with $10^8$ leaves and 9 levels, the average branching factor is only $10^{8/9} \cong 7.7$. So the height of the hierarchy should not be a limiting factor.

The scaffolds at the bottom of the hierarchy are inspired by the original work on molecular frameworks by Bemis and Murcko [16]. The middle of the hierarchy is based on ring topologies as described by Pollock et al [11]. The top levels are our design, further abstracting the idea of ring topologies. The hierarchy is a result of PubChem analysis and the process which lead us to this specific design of hierarchy is described in detail in the following section. Here, we limit ourselves to the introduction of the hierarchy.

**Fig. 1:**
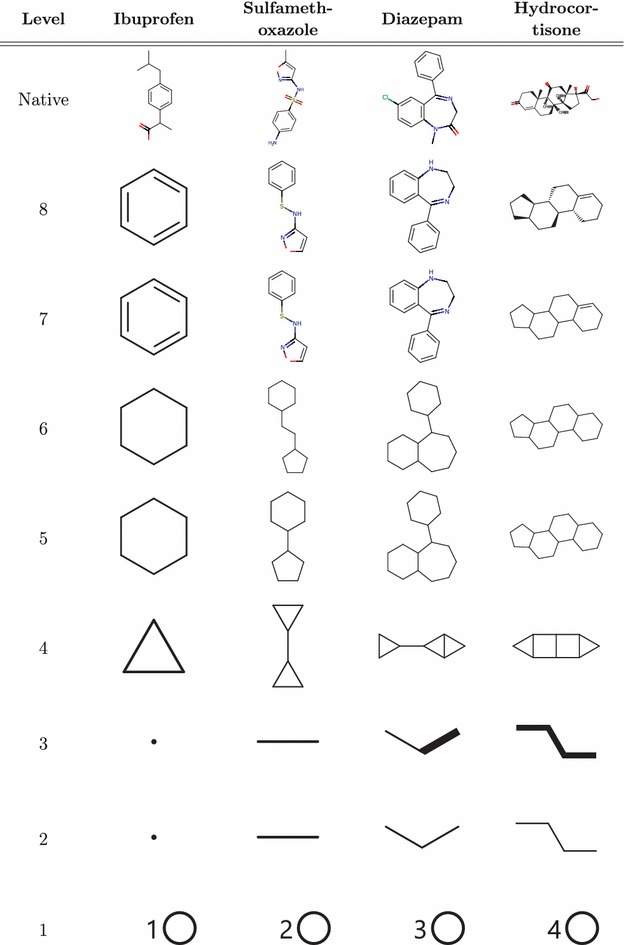
Scaffold hierarchy. Examples of a few well-known drugs and the scaffolds representing them in the scaffold hierarchy

Before introducing the hierarchy in detail we should stress out that Scaffvis framework allows modification of the hierarchy such as adding additional or modification of existing levels. This is described in Section 3.2 of Additional file 1. The following list describes the particular levels. For easier understanding of the transformations, four existing drugs together with theirs scaffolds on every level are depicted in Fig. 1. The drug molecules depicted are ibuprofen, sulfamethoxazole, diazepam, and hydrocortisone; the molecule data have been obtained from DrugBank [17], record ids DB01050, DB01015, DB00829, and DB00741 respectively.

*Level 8: Rings with linkers stereo* The bottom level contains all ring systems and linker atoms—i.e. it corresponds to molecule framework as defined above; however, at this level we conserve all the chemical stereo information that is left after the removal of side chains. To restate it another way, level 8 scaffolds are obtained from a molecule by the process of deleting sidechains, performing standard aromatization, neutralizing charge, removing explicit hydrogens and radicals, and discarding element isotope information.

*Level 7: Rings with linkers* The seventh level is identical to the bottom level except for that all the stereo information has been discarded. We have decided to remove the stereo information here because in the next step we are discarding information about chemical elements and in such reduced model the stereo information would be absolutely out of context.

*Level 6: Murcko rings with linkers* This level corresponds to the Murcko scaffolds, i.e. it is a skeleton of the original molecule with all of the chemical information discarded, atom connectivity being the only thing left. Therefore, to convert a level 7 scaffold to a level 6 scaffold we replace all elements by carbon and convert all bond types to single bonds. The number of nodes of the resulting graph is still equal to the number of (non-hydrogen) atoms in the base molecule framework.

*Level 5: Murcko rings*This level is obtained by removal of superfluous linker atoms—superfluous meaning atoms of degree 2. That means that we only leave branching linker atoms and replace all linker paths by a single edge. In this step, for the first time, the size of graph might be lesser than the size of the original molecule framework.

*Level 4: Oprea* The Oprea scaffolds are obtained by performing similar contraction on ring atoms as was done on linkers at level 5. In this process, we remove all remaining vertices of degree 2 by performing edge merging operation on them. The only exception being when both vertices’ neighbors are connected (i.e. we have a triangle), when edge merging would lead to a loss of the cycle. We obtain a minimum cycle topological representation of the original molecule.

*Level 3: Ring*Connectivity extended up to now, every vertex of a scaffold graph corresponded to an atom in the original molecule. However, on levels 2 and 3 the vertices do not correspond to atoms but to entire rings. Level 3 scaffolds are created from Oprea scaffolds in two steps.

First, the molecule is decomposed into rings. This by itself is a surprisingly difficult task. Two standard algorithms exist—SSSR (smallest set of smallest rings) and CSSR (complete set of smallest rings)—both giving very unexpected results in some edge cases. We decided for the CSSR variant as it seems to gradually replace SSSR as the method of choice in cheminformatics frameworks.

Second, having the set of small rings—the vertices of the new graph—we connect the rings that were connected in the original graph. We distinguish two types of connectivity:

1. Strong connectivity—when the two rings share an edge in the original graph.
2. Weak connectivity—when the two rings share a vertex in the original graph or when they are connected by an edge which is not a part of any ring, i.e. a “linker edge”, or when it is connected by a path formed by linker edges.

The rules for strong and weak connectivity try to reflect whether the connection of the original rings is rigid or flexible. Rings sharing an edge—fused rings—are considered to be rigid. Other connections are considered flexible/weak.

**Fig. 2:**
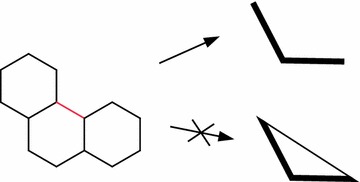
Level 3 exclusion rule. The red ring bond is excluded when calculating a ring connectivity scaffold; including the edge would form a triangle instead of a linear path

The restriction to linker edges is to prevent unexpected superfluous edges in the resulting graph. An illustration of why ring edges have to be excluded is provided in Fig. 2.

In the scaffold molecule representation, we model strong connectivity as bonds of order two (double bonds) and weak connectivity as bonds of order one (single bonds). In a visual representation, we show strong connectivity as bold bonds and weak connectivity as standard bonds.

*Level 2: Ring connectivity* A simplified variant of the ring connectivity scaffold takes out the distinction between the strong and weak connectivity, making all bonds single/standard, and distinguishing only between connected and not connected rings, with the same exceptions as at the previous level.

*Level 1: Ring count* On the top level we represent all molecules simply by their ring count. More precisely, that is defined as the number of vertices of the ring connectivity scaffolds at levels 2 and 3. Which is in turn equal to the number of rings in the CSSR decomposition of the Murcko scaffold or the original molecule.

*Level 0: Root* A single node at level 0 serves as the root of the scaffold hierarchy.

### Scaffold background extraction and analysis

As mentioned in the previous section, in Scaffvis we used the PubChem Compound database as our reference chemical space to be used as the background in the visualization. When choosing the reference database, an important criterion was that the database needs to be downloadable (ruling out ChemSpider [18]) and has free access (ruling out ChemNavigator). Another factor to consider was the context information that the chosen database would provide—as we want to compare frequency of a scaffold in a user dataset to the frequency of the same scaffold in the background database, a generic database of compounds (such as PubChem) would give a different picture than a database of known drugs (e.g. DrugBank [17]). But the decisive difference was the number of distinct scaffolds contained. The more scaffolds the background would contain, the larger portion of the chemical space we would visualized. The winner, in this respect, is the PubChem database as it is a large database and according to Wester et al. [19] it also has the highest scaffold diversity.

We should emphasize here that PubChem is not hard-coded in the solution and any other set could be used as the background. For example, more suitable choice of background for the drug discovery researchers would be DrugBank due to its narrower scope.^2^

Our hierarchy was developed by taking existing hierarchies and gradually adding new scaffold levels to reach a reasonable branching factor so that children of every node in the hierarchy could be reasonably displayed on a single screen. This should be satisfied on every level, including the root level—implying that the number of scaffolds at the top level (i.e. the children of the root) should be small enough. Before introduction of our hierarchy, the highest level ring topology-based scaffolds were the Oprea scaffolds. As it turned out, the number of Oprea scaffolds in PubChem Compound database was 138 thousand, making them very unsuitable candidates for the top level scaffolds. This led us to designing additional levels above the Oprea scaffolds.

The first attempt to abstract upon Oprea scaffolds was to represent every ring by just one vertex and to include information which rings are connected and how. Distinguishing two types of connectivity—a strong connectivity, when two rings share a common bond (graph edge), and a weak connectivity otherwise—brought down the count to 119 thousand, still infeasible for the top level.

In the next step, we disregarded the connectivity type, only distinguishing connected and not connected rings. The number was brought down more significantly, to 50 thousand. Which was unfortunately still too high.

In the last step, the ring connectivity information was removed completely, distinguishing the ring topologies only by ring count. This yielded an almost optimal number of 102 top level scaffolds.

Altogether, the described scaffold types form top four levels of the final scaffold hierarchy. Having the top levels ready, we designed the bottom four levels, this time taking most of the inspiration from the original article on molecular frameworks [16]. Again, in order to keep the number of children small, we aimed for as small steps between levels as possible; however, we have not subdivided the steps where it seemed chemically nonsensical. A notable example, where we opted for not-subdividing, was the step between level 7 (rings with linkers) and level 6 (Murcko rings), which discards bond multiplicity and heteroatoms (and other less important properties) all at once. That is a big leap in the information contained (leading to some level 6 scaffolds having excessive number of children), however, we could not see, how a smaller step could be made—for example discarding heteroatoms and keeping bond order or the other way around did not seem chemically justifiable and we did not find any examples in the literature where such partially simplified scaffolds would be used.

The above described process lead us to 9 level hierarchy as introduced in the previous section. Processing PubChem Compound with such hierarchy results in branching factors as shown in Table 1. Here, we divided scaffolds into four groups based on the number of children they have: optimal (1–100), good (101–400), large (401–1600), excessive (>1600). The limits come from the idea of organizing scaffolds into a square grid—100 scaffolds fitting into a $10\times 10$ grid, 400 fitting into $20\times 20$, and 1600 into $40\times 40$.

**Table 1:** Scaffolds by number of children, divided into four bins; the table shows how many scaffolds there are in each bin

Level	Number of scaffolds by branching factor							
	0–100		101–400		401–1600		>1600	
0	0	0.00%	1	100.00%	0	0.00%	0	0.00%
1	69	67.65%	15	14.71%	7	6.86%	11	10.78%
2	49,781	99.94%	28	0.06%	0	0.00%	0	0.00%
3	118,902	100.00%	0	0.00%	0	0.00%	0	0.00%
4	137,032	99.56%	455	0.33%	125	0.09%	21	0.02%
5	595,555	99.92%	472	0.08%	30	0.01%	1	0.00%
6	1,274,080	99.40%	5756	0.45%	1602	0.13%	350	0.03%
7	7,476,752	100.000%	35	0.00%	0	0.00%	0	0.00%

### Visualization methodology

There are several possible ways how to visualize a scaffold hierarchy. One possibility is to use the treeview-based visualization as was done in [10]. Another way is to create a hierarchical visualization akin to a zoomable geographical map, a concept most of computer users are familiar with. In order to do that, we need to be able to embed children of every scaffold into a plane, starting with the children of the root, i.e. the top level scaffolds. Embedding a set of scaffolds into a plain is a problem similar to traditional (non-hierarchical) visualization of a set of molecules. Having a similarity measure on scaffolds would allow to use the previously described methods (principal component analysis, multidimensional scaling or force directed layouts) to solve the problem. However, designing a common similarity measure for all levels of the scaffold hierarchy is quite challenging task. Since scaffolds are graphs, using the graph edit distance might look tempting. The problem is that two molecules with different scaffolds can easily have lower graph edit distance than two molecules sharing scaffold. This poses a wide array of problems for the visualization. Next, the distance should be transitive with respect to all the levels of the hierarchy. That is when scaffolds *A* and *B* share parent scaffold then *A* and *B* should be closer than any scaffold *C* which does not share parent with *A* and *B*. But that does not have to be true with respect to graph edit distance. Moreover, graph edit distance is an NP-hard problem and even existing heuristics are quite slow, or at least far from instantaneous on graphs of size of the scaffolds. On top of that, to calculate a distance matrix, the number of computations is quadratic to the number of displayed scaffolds. Some rare scaffolds in our hierarchy have over 16,000 children, which means that over 128 million distance computations would be needed for such scaffold to calculate its children’s pairwise similarity.

The solution used in Scaffvis is to display the scaffolds in a form of a tree map [20]. We chose the Squarified TreeMap algorithm [21] which aims at generating layouts in which the rectangles are depicted as squares as best as possible. When no user data set was loaded Scaffvis allows to browse the background hierarchy only. When a dataset is loaded, color and size of the tree map’s squares are used to encode the proportion of the user scaffolds in the background and user sets. Specifically, by default the area of a square is based on the relative frequency of given scaffold in the background hierarchy; the color is based on the frequency of the scaffold in the user data set.

### Implementation

The Scaffvis application is divided into three distinct parts—client, server and generator. The client application is a single-page web application offering the visualization functionality. The server provides support to the web application—mostly in the form of API to access the precomputed background hierarchy as well as the functionality of the chemical framework. The generator project is a simple command line application which serves the purpose of computing the background hierarchy. In this section we give a brief overview of the implementation, to read more detailed description, including application design considerations, see Additional file 1.

The client–server model was chosen due to relatively big size of the background hierarchy with respect to current browser capabilities (about 800 MB of data for the PubChem database). Therefore we store the hierarchy on the server and the client application accesses the required data through an API. Second reason was the need to perform relatively complex chemical calculations. To our knowledge, there is no suitable JavaScript cheminformatics framework available. There exist cheminformatics browser components by ChemAxon (such as Marvin JS), but none of these components fulfilled our needs of complex molecular transformations when dealing with scaffolds. Finally, we settled for the ChemAxon JChem toolkit and implemented all the chemical calculations on the server side. The implementation is very straightforward and is shared between the server and the generator. The client application accesses the needed chemical calculations through a high-level API.

All parts of Scaffvis (client, server and generator) are written in Scala programming language [22]. This fits well with our choice of ChemAxon JChem as our cheminformatics toolkit as both run natively on the JVM. The client uses Scala.js [23]—a Scala to JavaScript compiler. React^3^ is used as a UI library through the scalajs-react^4^ wrapper. React is complemented by Diode^5^ for application state management. The server is based on the Play Framework.^6^ To store the scaffold hierarchy we used the MapDB^7^ database engine.

## Results

The application, Scaffold Visualizer (Scaffvis), has been published under the GPLv3 license and its code is freely available at https://github.com/velkoborsky/scaffvis. Scaffvis can either be installed on a dedicated server or run locally. The installation guide is available on-line. A running instance is available at http://scaffvis.projekty.ms.mff.cuni.cz/. The following sections covers separately results describing the background hierarchy of the PubChem database built using the generator and the visualizer itself.

### PubChem scaffold analysis

The scaffold hierarchy of an arbitrary dataset can be created using the generator which allows to run predefined *tasks*. Currently, the main purpose of the generator is to generate the background hierarchy from the PubChem Compound database, but with only minor modifications any other custom background compound set can be used. The generator can also be used to run arbitrary analytical task over the scaffold hierarchy, allowing for customized and targeted exploration of the scaffold space. Such customized tasks have been used during the process of designing the hierarchy and the final visualization. Also all the data presented in this section have been obtained using the task which can be downloaded from the Scaffvis repository (*HierarchyStatistics.scala*)—allowing to recalculate the statistics using more recent data or to modify the task to extract additional statistics one would be interested in. Details about the process used to obtain the hierarchy can be found in the Additional file 2.

All the presented data are based on PubChem Compound database as of July, 2016, containing 91.4 million molecules. The generated scaffold hierarchy is available as a CSV files and can be accessed from the Scaffvis repository.

The total number of scaffolds in the hierarchy is 19.5 million. A break down by levels is provided in Table 2.

**Table 2:** Number of scaffolds per hierarchy level

Level	Name	Number of scaffolds
0	Root	1
1	Ring count	102
2	Ring connectivity	49,809
3	Ring connectivity extended	118,902
4	Oprea	137,633
5	Murcko rings	596,058
6	Murcko rings with linkers	1,281,788
7	Rings with Linkers	7,476,787
8	Rings with Linkers Stereo	9,867,182
	Total	19,528,262

The branching factor of scaffolds in the tree was already analyzed in the section introducing the scaffold hierarchy and the results can be found in Table 1. Another view at the same distribution can be gained through percentiles which provides Table 3. It can be immediately noticed that all numbers for level 0 are identical. That is due to level 0 being the root level which contains only a single scaffold, the root scaffold, that has exactly 102 children (distinct ring count scaffolds). Therefore, the average, the minimum ($\mathrm {P}_0$), and the maximum ($\mathrm {P}_{100}$) number of children, as well as all other percentiles, are identical and equal to 102. Generally, we can see that the distributions are skewed—there are lots of scaffolds with small amount of children and then small amount of scaffolds with high number of children.

**Table 3:** Number of children per scaffold—distributions by level; the table shows how many children the scaffolds at each level have—for example the scaffolds at level 4 have on average 4.33 children each, also at least $90\%$ of level 4 scaffolds have at most 5 children

Level	Number of children per scaffold percentiles									Mean
	$\mathrm {P}_{0}$	$\mathrm {P}_{10}$	$\mathrm {P}_{25}$	$\mathrm {P}_{50}$	$\mathrm {P}_{75}$	$\mathrm {P}_{90}$	$\mathrm {P}_{95}$	$\mathrm {P}_{99}$	$\mathrm {P}_{100}$	
0	102	102	102	102	102	102	102	102	102	102.00
1	1	1	4	29	199	1660	3396	5083	5180	488.10
2	1	1	1	1	2	4	7	25	188	2.38
3	1	1	1	1	1	1	2	4	35	1.16
4	1	1	1	1	2	5	9	43	3666	4.33
5	1	1	1	1	1	3	6	20	2206	2.15
6	1	1	1	1	2	6	11	60	16,755	5.83
7	1	1	1	1	1	3	3	5	363	1.32

To get insight on what are the most common scaffolds in PubChem we analyzed each level separately and extracted ten most common scaffolds at each level.

Level 1, the ring counts, is summarized in Table 4. The molecules with small number of rings are by far the most frequent. The corresponding scaffolds have small number of children, which is expected, as there are only a few ways how to connect a small number of rings. For higher ring counts, the number of children grows rapidly. The table also shows one anomaly, which is the number of children of the node representing two-ring scaffold. Since there is only one way to connect two rings, the expected number of children would be one, but the table shows two. The reason is that although it is quite rare (less than 0.1% compounds) there exist disconnected compounds in the PubChem Compound database. Thus, the second topology corresponds to two disconnected rings.

**Table 4:** Top ten most frequent ring counts in PubChem Compound database

Ring count	Frequency in PubChem (%)	Number of children
0	3.12	1
1	14.01	1
2	30.74	2
3	25.68	4
4	16.03	10
5	6.59	27
6	2.10	97
7	0.69	360
8	0.37	1044
9	0.21	1948
Total	99.52	

**Fig. 3:**
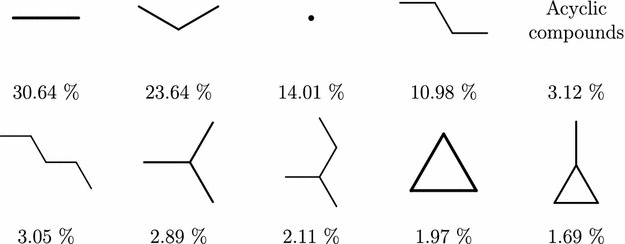
Level 2 PubChem scaffolds frequency. Most common ring connectivity scaffolds (level 2) and their frequency in PubChem compound database

The top ten most common level 2 scaffolds are depicted in Fig. 3 accompanied by their frequency. We see that, similarly as at level 1, the top 10 scaffolds cover a large fraction of the compound database—$94.10\%$. The branching factor in this level is low with all of the scaffolds having at most 12 children.

**Fig. 4:**
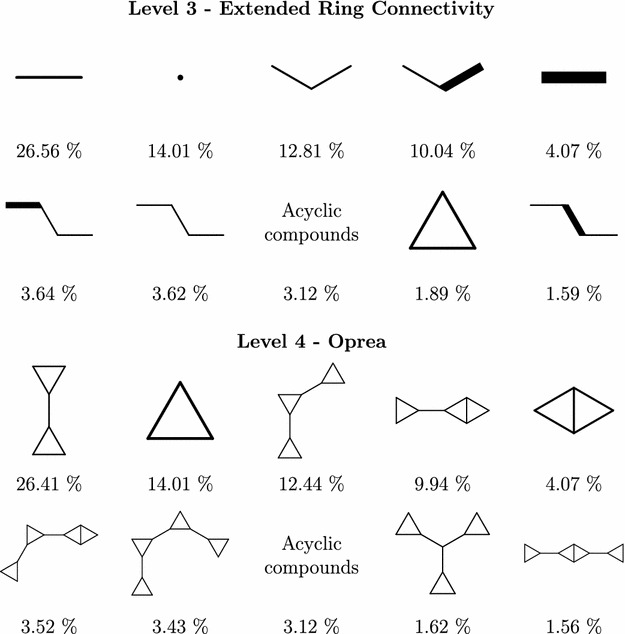
Level 3 and 4 PubChem scaffolds frequency. Most frequent scaffolds at levels 3 and 4 correspond to each other

An interesting phenomena can be observed on levels 3 and 4 (extended ring connectivity and Oprea). On these levels, the most common scaffold cover very similar portions of the PubChem compounds – $81.35\%$ in case of level 3 and $80.12\%$ on level 4. Moreover, the ten most common scaffold correspond to each other, as can be seen in Fig. 4. Not only the order of the corresponding scaffolds is the same on both levels, even the fractions of molecules they represent are very similar. So although most of the depicted level 3 scaffolds have more than one child, in every case one of the children is disproportionately more frequent than the others, and belongs to the top 10 scaffolds on level 4. This suggests that level 3 and level 4 scaffolds, although looking very different, convey very similar information.

**Fig. 5:**
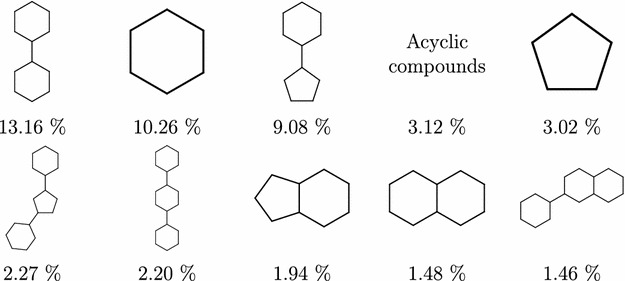
Level 5 PubChem scaffolds frequency. Most common Murcko Rings (level 5) scaffolds—graph frameworks with contracted linkers

**Fig. 6:**
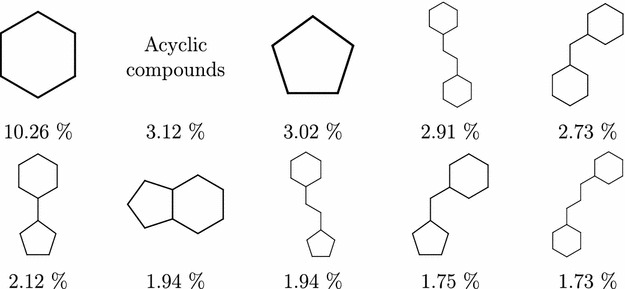
Level 6 PubChem scaffolds frequency. Most frequent Murcko rings with linkers (level 6) scaffolds—graph frameworks with linkers preserved

Going further down to the Murcko graph frameworks (level 5), the top 10 scaffolds shown in Fig. 5 still cover almost half of the PubChem compounds ($47.98\%$); that falls rapidly on level 6, where the top 10 scaffolds (Fig. 6) cover only $31.51\%$.

**Fig. 7:**
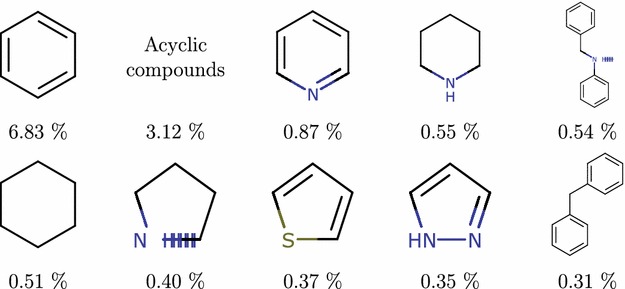
Level 7 PubChem scaffolds frequency. Most rings with linkers (level 7) scaffolds and their frequency in PubChem compound database

Finally, on molecular framework levels 7 and 8 the top scaffolds are identical. Most common level 7 scaffolds can be seen in Fig. 7. Their frequencies on both levels are almost identical as well—with $13.85\%$ total for level 7 and $13.82\%$ for level 8. Although under $14\%$ might seem like a low number, compared with the fractions before, there are 7.5 million scaffolds at level 7 and almost 10 million scaffolds at level 8; in that context, a single scaffold covering around $0.5\%$ is still very significant. There is also a noticeable predominance of aromatic rings over non-aromatic ones.

### Scaffold visualizer

In the previous section we described the generator and results it yields when run against the PubChem Compound set. Here we describe the capabilities of the scaffold visualizer. Scaffvis is a client-sever application which enables to interactively explore molecule data sets based on the scaffold hierarchy. Molecular formats supported in Scaffvis are derived from capabilities of the ChemAxon JChem toolkit, our cheminformatics framework. JChem supports both SDF and SMILES input formats, including gzipped versions of these formats. This is especially useful when dealing with large data sets. Here we need to emphasize that even for small gzipped files the loading of a dataset might require substantial time—proportional to the number of molecules in the dataset. When no dataset is loaded, the visualizer allows to browse the background scaffold hierarchy which was analyzed in the previous section. The capabilities of the visualizer summarizes the following list. Scaffvis allows to:

- browse the scaffold hierarchy as a zoomable tree map—explore molecular scaffolds in the underlying PubChem Compound database and their frequency;
- import an existing data set in all common cheminformatics file formats (SDF/Molfile, SMILES, InChI, CML, and more);
- display imported compounds in the tree map;
- colorize the tree map according to scaffold frequency in user data set, in PubChem, frequency in user data set relative to frequency in PubChem;
- change the used color gradient;
- base tree map element sizes either or frequency in data set or on frequency in PubChem;
- apply logarithmic transformation to the sizes or the color source values;
- select molecules by scaffold or directly;
- search for molecules;
- filter molecules based on the selection, the search filter and the current subtree;
- browse the filtered molecules in a pageable list;
- display scaffolds as a list instead of a tree map;
- sort the scaffold list by frequency in data set, in PubChem, or lexicographically;
- export the molecules based on the selection, search filter and current subtree;
- export scaffolds on the current level;
- save current data set including the computed scaffolds and selection;
- bookmark or link the current position in the scaffold hierarchy.

**Fig. 8:**
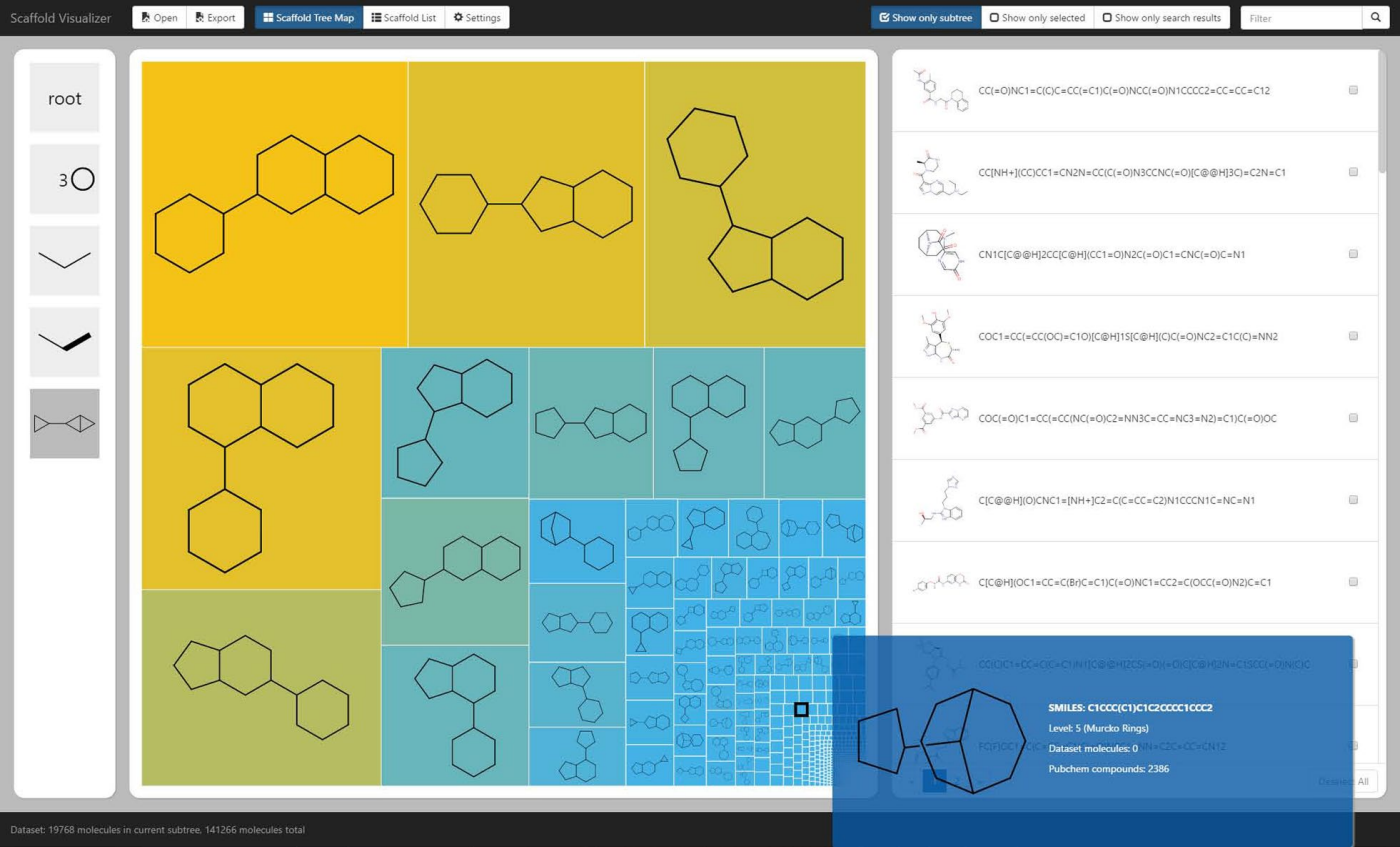
Scaffvis main screen. Main screen of the scaffold visualizer

The user interface of the visualizer shows Fig. 8. The interface consists of several basic components—in the center of the image, from left to right are the breadcrumb navigation, the scaffold box and the molecule box. Above and bellow that are the header and the footer. In the lower right corner is displayed the tooltip. Not shown in the picture are three forms—Load data set form, Export data form and Settings form. Here, we only briefly describe the most important components of Scaffvis, namely the scaffold tree map, scaffold list and molecules list, while the description of all the remaining components can be found in Additional file 3.

The scaffold tree map is the main component of the hierarchical visualization. It displays a set of scaffolds, each scaffold represented by a rectangle which holds the picture of the scaffold. The rectangle’s area is by default based on the relative frequency of the scaffold in the background hierarchy; the color is based on the frequency of the scaffold in the user data set. Clicking a scaffold selects or deselects molecules corresponding to that scaffold. On mouse wheel scrolling or double click the tree map is zoomed in (navigating to the scaffold under the mouse pointer) or zoomed out (navigating to the current scaffold’s parent). Moreover, upon hovering the mouse above a scaffold the tooltip is shown providing detailed information.

Analogous functionality to the tree map is available through the scaffold list component. The form is, however, different. The scaffolds are shown as a list, sorted lexicographically or by their frequency in the user data set or in the background hierarchy. Each item in the list contains a small image of the scaffold, its name, level in the hierarchy and the number of molecules in the user data set and in the background hierarchy that correspond to the particular scaffold. A button for “zooming in” the scaffold is present. Clicking the scaffold selects the corresponding molecules, same as in the tree map.

The third core component of Scaffvis is the molecules list. The molecules list is empty until a user data set is loaded. After that, it shows the list of loaded molecules. By default, the list is restricted to the molecules in the current subtree, i.e. the molecules that correspond to the current scaffold. The list can also be restricted to show only the selected molecules or to only show search results (as long as a search query is entered). All the three filters can be toggled using the buttons at the right side of the header. The search query can be entered in the header above the molecules list. For each molecule a picture is shown, together with its SMILES string and its name and comment (as long as they were present in the input). Clicking a molecule selects it. The molecules are shown by pages consisting of 50 items. The total number of pages is not precalculated to avoid evaluation of the filters on huge data sets, i.e. only the next page and the preceding pages are available in the pager.

#### Performance

The application is able to handle relatively large data sets, which is especially important considering the size of data sets used in context of high throughput screening methods. The exact size of data set that can be loaded depends on the used browsers and even on the data itself. The most significant limitation is the amount of memory available to the browser JavaScript interpreter. Using the Google Chrome browser, version 51, 64-bit, a dataset of 500 thousand drug-like molecules from ZINC database has been opened and the application performed well. On the other hand, in case of large datasets the loading of the dataset itself can be relatively time consuming. On our reference 4-core 4-GHz CPU, the server is able to process about 1000 molecules per second—meaning that the 500 thousand molecules large dataset takes almost 10 minutes to load. The processing time is linear to the size of the input. Of the processing time, it takes about 25% to load the molecules from the input format and to calculate their canonical SMILES inner representation; the other 75% of time is spent calculating the scaffolds and searching for them in the scaffold database.

To decrease the dataset loading time, two approaches have been employed. One being asynchronous loading of molecule images—the molecules are only rendered when they are to be displayed. And as the molecule list component only displays 50 molecules at time, only a small number of images is required at any moment. Another approach to reduce to loading time is the implemented binary export format—the molecules can be saved together with their generated scaffolds and such data set can be opened without even being sent to the server, reducing loading time from minutes to seconds for the largest data sets. However, this approach only helps when a data set is used repeatedly while the first processing always takes the full time.

## Conclusions

We have designed and implemented a new approach for scaffold-based hierarchical visualization of molecular datasets. The approach is based on two key components—a ring topology-based scaffold hierarchy providing a background, i.e. context and reference, for a zoomable tree map visualization method.

The proposed hierarchy aims to provide a stable background for visualization. Each molecule is classified by exactly one scaffold on each level of the hierarchy. The scaffold definitions are based on existing concepts of molecule frameworks and ring topologies; however, the ring connectivity scaffolds are a new type of scaffolds introduced by this work. By default, the method uses PubChem, the largest chemical database available, as the background scaffold hierarchy. The PubChem scaffold hierarchy is available in the Scaffvis repository together with the source codes allowing to calculate it on commodity hardware in the course of a few hours.

The visualization itself was implemented into Scaffold Visualizer (Scaffvis)—a client-server visualization application allowing interactive exploration of user data set in a form of a zoomable tree map. Scaffvis is a web browser tool, which is accompanied by a server counterpart that provides the background data and the chemical functionality. Although web-based application, Scaffvis allow to handle data sets containing hundreds of thousands of compounds.

## Availability and requirements

- Project name: Scaffvis
- Project home page: https://github.com/velkoborsky/scaffvis
- Operating system(s): Platform independent
- Programming language: Scala
- Other requirements: JVM + Chemaxon JChem
- License: GNU GPLv3

## Additional files

**Additional file 1.** Scaffvis architecture. Detailed description of Scaffvis architecture, application design choices and employed technologies.

**Additional file 2.** Generating the PubChem background hierarchy with Scaffvis Generator. Description of tasks used for generating the PubChem background hierarchy.

**Additional file 3.** Scaffvis interface components description. Detail description of interface components.

## Footnotes

1 From that we can observe that a molecular graph belongs to a particular topology if and only if it can be obtained using a series of edge subdivisions.

2 Scaffvis repository includes a tool allowing to extract the hierarchy from an arbitrary set of molecules with only minimal modifications (changing the input format).

3
https://facebook.github.io/react/.

4
https://github.com/japgolly/scalajs-react.

5
https://github.com/ochrons/diode.

6
https://www.playframework.com/.

7
http://www.mapdb.org/.
