# Supplementary material for: Scaffold analysis of PubChem database as background for hierarchical scaffold-based visualization
Source: J Cheminform. 2016 Dec 29;8:74. doi: 10.1186/s13321-016-0186-7 (PMC5199768; doi:10.1186/s13321-016-0186-7)
Supplement: Supplementary file 1 — Additional file 1. Scaffvis architecture. Detailed description of Scaffvis architecture, application design choices and employed technologies. [file 13321_2016_186_MOESM1_ESM.pdf]

Scaffvis implementation details

Jakub Velkborsky, David Hoksza

Here we describe the architecture and selected implementation details of Scaffvis. We discuss the reasons behind why the particular architecture has been chosen. We follow up by a list of selected technologies being used. And then we describe the implementation itself, especially in respect to modifiability.

1 Architecture

Scaffvis is divided into three distinct parts - a *client*, a *server* and a *generator*. The *client* application is a single-page web application offering the visualization functionality of our solution. The *server* provides support to the web application - mostly in the form of API to access the precomputed background hierarchy as well as the functionality of the chemical framework. The *generator* project is a simple command line application which serves the purpose of computing the background hierarchy.

This architecture is not an arbitrary but follows from the underlying problem that is being solved. The motivation to provide the visualization in the form of a web application stems from usability reasons, such as low access barrier, multiplatformity or even the potential for a later third-party integration into a larger chemical web project. So the obvious solution would be to implement the whole solution as a standalone client-side application. And we would have done so, had it been viable.

1.1 Client-server model

There were two separate reason why a standalone client-side web application was unfeasible and a server component was needed. The first reason was the prohibitive size of the background hierarchy used, the second reason was the difficulty of performing chemical calculations on the client side.

First, to the background hierarchy size. The background hierarchy, calculated up to its specification described in section **??** and based on the PubChem Compound database, contains over 14 million unique scaffolds (as of July 2016). For each of the scaffolds we need to store at least its ID, ID of its parent and the scaffold itself - in our case we used the canonical SMILES representation, which is very space efficient, but it was still 45 characters long on average. Additionally, we would like to at least reference children from the parents, which is in total another ID per record. Although it is a very crude estimate (in reality we store more information and have secondary indices), it is 14 million records, each 57 bytes on average (3×4 bytes for IDs and 45 bytes for SMILES), totaling 800 megabytes of data. That is not only unfeasible to bundle with the application (even when compressed), but it is also on the verge of memory limit of modern browsers[1]. There might be ways to work around these problems but there was no sufficient motivation for to attempt such extreme feat.

In our design, we store the hierarchy on server and the client application accesses the required data through an API.

Second, to the topic of preforming in browser chemical calculations. To our knowledge, there is no JavaScript cheminformatics framework available. There are some cheminformatics browser components by ChemAxon (such as Marvin JS[2]), which was another reason why closely explored ChemAxon product portfolio (which in the end let to the choice of ChemAxon JChem), but none of these components fulfilled our needs (described in the section **??**).

We also experimented with using RDKit or Open Babel, transpiled from C++ to JavaScript using Emscripten [6], a compiler plug-in for LLVM [3]; following a helpful series of blog articles on this topic written by Noel O'Boyle [5]. On the one hand, we managed to create a functional prototype, on the other hand, there was very significant added complexity compared to using these frameworks directly and the potential advantages (saving server CPU time) were not worth so significant complication in the application design.

Finally, we settled for the ChemAxon JChem toolkit and implemented all the chemical calculations on the server side. The implementation is very

[1]For example the Google Chrome version 51.0 (32 bit) has memory limited to 756.3 MB per page. This information is based on the `window.performance.memory.jsHeapSizeLimit` property accessible from browser's console.

[2]`https://www.chemaxon.com/products/marvin/marvin-js/`

straightforward and is shared between the server and the generator. The client application accesses the needed chemical calculations through a high-level API.

Still, all logic that could be reasonably implemented on client-side, is implemented on the client-side, thus resulting in a thick client - thin server architecture

1.2 Preprocessing the hierarchy

The PubChem Compound database in compressed SDF format is 61.5 gigabytes large, as of time of writing (July 2016). It is obviously virtually impossible to use this form directly for any kind of online scaffold analysis. Thus, the data have to be preprocessed. This processing of a source background database into a form suitable for online analysis is the primary purpose of the generator project. For each molecule scaffolds on all levels are computed, assigned unique numerical identifiers and these identifiers are stored to a tree allowing for instant retrieval of scaffold's parent and children. During the development, we have also used the generator project to run all kinds of ad-hoc analytical queries on the generated database - a practice that we can only recommend.

2 Technologies Used

Having described the three main blocks of the application, let us offer a list of some technologies used in their implementation as well as our experience with them. This list of technologies is by no means exhaustive and the technologies are listed in no particular order.

Scala All parts of the application - the generator, the server and the client - are created using a single programming language - the Scala programming language [4]. Scala is natively a JVM language - that is a language that runs on the Java Virtual Machine (JVM). Scala has been created by Martin Odersky, who is also the author of the current reference Java compiler and who directly influenced addition of generic types to Java[3]. Scala is a language

[3]Odersky created a language called Generic Java which was a superset of the Java language with added support for generic types. The design has been used by Sun Microsystems, Inc. as the basis for generics support in the Java.

designed to be compatible with Java, it is fully interoperable with Java (which especially implies that all existing Java libraries can be used) and it can almost serve, as a drop in replacement for Java as it is possible to write very Java-like code in Scala. But Scala also has numerous advantages compared to Java - it has been designed as a functional language, it has a great standard library, it is very extensible and has a great type system, which altogether allows for very succinct code to be written; and shorter code arguably leads to better maintainability and less bugs - at least such has been the author's experience with the Scala language.

We were bound to choose a JVM language due to our choice of ChemAxon JChem as our cheminformatics toolkit. We chose Scala over Java partially because of the advantages listed above and partially because Scala can also be used as a language for browser development, allowing us to use only one language all across the application, simplifying the development and increasing the maintainability significantly.

Scala.js Scala.js [2] is a transpiler from Scala to JavaScript. It allows browser applications to be written in Scala. Due to resemblance between Scala and JavaScript - both being objective and functional C-family languages - the Scala code is very close to the equivalent JavaScript code; that specifically allows most of the documentation and resources available for JavaScript programming to be used. Scala also has a significant advantage over JavaScript in the form of statical type checking.

We implemented a prototype client application in both - Scala.js and JavaScript. Comparing both implementations, we chose to use Scala.js for our project. The advantages were many. Using Scala on both - the server and the client - significantly simplified interoperability between them - allowing for example the same data structures to be used on both sides - again reducing code duplication and improving maintainability. The static type checking, that Scala has over JavaScript, also prevented innumerous errors, simplifying the development. Finally, it was also very comfortable to only use one language - reducing mental context switching. Amongst the disadvantages, the most significant disadvantage we came upon was that the transpiled code is very hard to debug in the browser. Another slight disadvantage could be that wrappers have to be written around native JavaScript libraries, but it was not a problem in our case as wrappers for all the libraries we used already existed.

Overall, our experience with Scala.js was great and we would recommend it for any comparable project.

React React[4] is a JavaScript library for building user interfaces, which we used in the client application. React breaks the application into distinct composable components. Each component gets a set of properties and can also have an inner state (which however is discouraged). A component also has a render function that based on the properties and state renders the component into virtual DOM (an equivalent of standard browser DOM, which is however a lot faster to update). React then automatically manages rendering components to virtual DOM (when properties or state are changed) and also synchronizes the virtual DOM to browser DOM.

We used React through scalajs-react[5] wrapper by David Barri which provides a type safe API to access React. We were satisfied with both - React and scalajs-react.

Diode Diode[6] is a Scala.js library by Otto Chrons, used for managing application state. The state is a collection of immutable objects. Updates of the state are performed exclusively by a designed hander, based on a stream of actions. Diode facilitates to build an application based on so called *unidirectional data flow* - a paradigm commonly used with React; in simple terms that means that the application has a central state (a single source of truth), this central state is passed to React components (in form of *properties*) and based on the state the components are rendered. The components may dispatch actions, for example on user interaction. These actions are then processed by the central handler, leading to the state being updated - and the cycle is closed.

The unidirectional data flow architecture plays well with a functional language, such as Scala, with emphasis on immutable data collections. The Diode library worked well for us and can be recommended.

MapDB MapDB[7] is a simple embedded Java database engine by Jan Kotek. It provides implementation of off-heap and on-disk data structures

[4] `https://facebook.github.io/react/`

[5] `https://github.com/japgolly/scalajs-react`

[6] `https://github.com/ochrons/diode`

[7] `http://www.mapdb.org/`

such as B-trees and hash maps, without the added complexity of using a fully-featured database system. We use MapDB to store intermediate results during processing of the background hierarchy as well for storage of the final result. The server also queries MapDB when processing user requests and uses in memory MapDB database as an image cache.

Overall our experience with MapDB was good. Unfortunately, during development we managed to get the database into an inconsistent state several times during a forced JVM shutdown - despite using transactions and a write-ahead log. So we are not convinced by MapDB's fault tolerance. Also, the performance is sometimes slower than we would imagine, especially for the purposes of the server component. Compared to the on heap collections in the Scala standard library, MapDB has been more than 100 times slower for the queries used by the server - even for repeated queries with supposedly warm disk cache. These queries, however, are not performance limiting for the server and so we kept using MapDB - to keep RAM requirements low. At any rate, after our application has been finished, a new major version of MapDB has been released, that is a total rewrite from the scratch; therefore, our experience is probably not applicable to the new version.

Play Framework A lightweight web framework used as a basis for our server component. Play framework is based on Akka[8], is stateless, asynchronous, has a very clean design and many other features. For us, however, the most important feature was the convenience that Play brings into the development process - it is trivial to create a new project, it is obvious how to extend it a the documentation is very good. Moreover, Play Framework comes with a bundled JBoss Netty web server, which can be used in both development and production. Running the code in development mode comes with built in hot-reloading and detailed and useful error messages. The production mode brings increased speed and secruity. Play Framework can also automatically generate a distributable package in the form of a portable multiplatform ZIP file; alternatively, native packages can be generated, such as Linux packages (DEB, RPM), Microsoft Installer (MSI) or OS X disk images. These distributable packages contain all required dependencies, including the

[8]Akka is "a toolkit and runtime for building highly concurrent, distributed, and resilient message-driven application on the JVM", as described by Akka's creators. Akka enables actor-based concurrency, an approach inspired by the Erlang programming language's library.

Netty web server, excluding only the Java Virtual Machine.

Overall, the Play Framework is both easy to use and scalable, making in suitable for all stages of an application - from an early prototype to a a final product. Web applications based on Play Framework are also easy to distribute and run for the end users, which was also an important concern for us.

3 Implementation overview

In this section we give an overview of how the application code is structured. We also describe some implementation details, where they are important or interesting, but opt to skip them elsewhere to keep the size of this section within limits. We also pinpoint parts of the code, which are suitable for customization.

3.1 Project structure and build process

The application is divided into multiple *projects*, which are defined and brought together using a single sbt[9] build definition.

Each of the the basic components of the application - server, client and generator - resides in its own likewise named project. All three projects can be built simultaneously using sbt. The *generator* and *server* projects are compiled into JVM bytecode, while the *client* project is transpiled into optimized JavaScript (JS) code, which is then passed to the *server* as a static resource to be served to the clients.

The code that is shared between the client and the server (e.g. the API interface) or between all the tree projects (such as basic data structure definitions) resides in a separate project called *shared*; this project is then both compiled to JVM bytecode and transpiled into JS. The code which is shared only between the server and the generator resides simply in the generator project - fragmenting the code in another shared project seemed to be more confusing than helpful - the generator project is simple enough, uses all the

[9]The name "sbt" stands for Scala Build Tool (`http://www.scala-sbt.org/`). It is an open source build tool similar to Maven, Ant or remotely to Automake. Sbt is the tool of choice for Scala projects and is sometimes used even for Java project - especially those built on the Play Framework.

shared code (in fact by definition), meanwhile the amount of generator specific code is quite limited (mostly the processing scripts) and last but not least the generator forms a meaningful stand-alone project - large amount of analysis behind this thesis has been done with the generator alone, before even the server project was started.

3.2 Generator

The generator project has three major functions - integration with the cheminformatics toolkit, processing of the PubChem Compound database into the background hierarchy, and providing access to the processed hierarchy (for the server or for user defined tasks). The functionality is divided into the following packages:

chemistry The *chemistry* package implements all the functionality based on the used ChemAxon JChem toolkit. The toolkit is not directly referenced anywhere else in the whole project and so it should be in the ideal case possible to replace the JChem toolkit by any other just by reimplementing the methods in this package. Major part of the implemented functionality are the scaffold transformations for our scaffold hierarchy. Besides that there are also thin wrappers to the chemical format conversion functionality (parsing an input stream, converting an individual molecule from and to SMILES), the imaging functionality (converting a molecule to its SVG representation) and some other minor functions.

hierarchy Next is the *hierarchy* package where our scaffold hierarchy is defined together with a generic interface to access a generated hierarchy. Notable is the *HierarchyTransformations* object[10] where the chemical transformations used to obtain scaffolds from molecules are defined. In case there was a need to use a different hierarchy, this is the file to be modified. Specifically, to modify the existing scaffold types, one needs to specify level operations in the *levelTransformations* field of the *HierarchyTransformations* object and subsequently implement them in the *ScaffoldOps* object of the *chemistry* package. Another file that would likely have to be defined is the *HierarchyLevels* object, which

[10] It indeed is a singleton object, not a class. Scala provides support for easy definition of singletons as one of its numerous advantages over Java.

defines the names of the hierarchy levels, and which resides in the shared project as it is shared by the client application.

tasks The *tasks* package contains definitions of all the processing tasks performed by the generator - most importantly the tasks used to obtain the background hierarchy from the PubChem Compound database. This is also a suitable place for user defined ad-hoc analytic tasks on the processed hierarchy - the *HierarchyStatistics* task may serve as an example.

processing The *processing* package contains some helpers used by the aforementioned tasks. The *ProcessingHelper* object provides a generic method of processing one key-value map into another providing parallelization, crash recovery (if the processing is interrupted unexpectedly, the progress is not lost), computation timeouts (when the cheminformatics toolkit hangs on one of the tens of millions of molecules), error logging, and so forth. This package is not expected to be modified.

stores The *stores* package encapsulates all the functionality related to the underlying MapDB database which is used to store the final generated background hierarchy as well as the intermediate results. Replacing the MapDB with another database, if it was so desired, should be quite straightforward by reimplementing this package and accommodating the few places in code that directly use the *org.mapdb.BTreeMap* interface.

configuration The only one left is the *configuration* package whose sole purpose is to provide paths to the database files for the *stores* package and a path to the PubChem Compound files to be processed - used by one of the tasks.

Last there is *Application* object which allows for the tasks to be called from the command line.

3.3 Server

Second of the three main projects is the *server*. The most important functionality of the server lies in the API that it provides to the client application. Through the API, the client application accesses the background hierarchy data as well as the functionality of the cheminformatics toolkit.

The server is based on the Play Framework, already described above. Besides processing the API calls, the Play Framework is used for some additional minor functionality such as, but not limited to:

- serving the client application to the client, using a simple template page;
- compilation of the SCSS stylesheet and providing the compiled CSS to the client, including the Bootstrap CSS library we use;
- serving other static resources such as fonts;
- routing, dependency injection, configuration;
- and of course the functionality described in the section 2.

The functionality is again divided into a few packages:

components The first package, *components*, contains two components - *ScaffoldHierarchyComponent* and *SvgComponent*. The scaffold hierarchy component provides, as many would guess, access to the background hierarchy. However, it is also able to calculate scaffolds for a given molecule and return its position in the hierarchy - i.e. it also contains a part of the mentioned functionality based on the cheminformatics toolkit. The SVG component provides SVG pictures of molecules and scaffolds; the scaffold pictures are also being cached using a LRU[11] cache based on an MapDB in memory hash table. The cache size can be specified in the configuration file.

controllers The *controllers* package mostly provides wiring. The most interesting pieces of technology used here are probably Autowire and uPickle. Both are libraries by Li Haoyi[12], an influential member of the Scala.js community. The uPickle library provides conversion between in memory data objects (defined in the *shared* project) and their JSON representation. Autowire provides routing Ajax/RPC calls to the API methods. What is really interesting is that the same two libraries are used in the client application. Consequently, the serialization and deserialization is almost transparent (yielding equal objects); and the RPC

[11] Least Recently Used - discards the least recently used items when the cache is full.
[12] `https://github.com/lihaoyi`

calls behave almost like standard method calls, including typesafety. The main difference is that the result of such call is wrapped into a future[13], which is however most convenient.

services Last is the *services* package containing the implementation of the API methods, based on the two components from the *components* package. The API interface itself is defined in the *shared* project, as it is also used by the client application. The functionality provided by the API is the following: 1) read access to the background hierarchy (for a given scaffold, fetch its children or ancestors); 2) providing SVG pictures (given a scaffold or a molecule, return its SVG depiction); and 3) processing user input (read molecules from an uploaded file, calculate their scaffolds and return the position in the background hierarchy). User input is processed in parallel, even for one user, as the processing is computationally quite expensive (more on that later) and the number of users using the application concurrently is expected to be low.

3.4 Client

The *client* project is a web browser application which provides the visualization functionality - it takes a set of molecules as an input from user and displays these molecules in the context of the background scaffold hierarchy. The molecules are shown in a form of a pageable list. The scaffolds are, by default, shown as a tree map, in which the size of every rectangle corresponds to frequency of the scaffold in PubChem Compound database and the color of a rectangle corresponds to the count of molecules with this scaffold in user data. Both are configurable, so for example the size can be made dependent on the dataset and color on the PubChem. Alternatively, scaffolds can also be shown as a sortable paged list. The molecules are also selectable and basic search functionality is also included.

The whole *client* application is built around the React library, which is an UI library, described in short in section 2. As the code is written in Scala, scalajs-react[14] wrapper around React is used, which provides a type safe access to the React library. Moreover, scalajs-react also provides a

[13] A *future* is a read-only placeholder for a future value used to await blocking. A *future's* value can be accessed providing a callback. Consult `http://docs.scala-lang.org/overviews/core/futures.html` for detailed information.

[14] `https://github.com/japgolly/scalajs-react`

routing component which is used to handle the application URLs (the URL always points to the currently explored scaffold and is stable - i.e. can be bookmarked or shared).

Accompanying scalajs-react is the Diode library, also described in section 2. Diode is used to store the state of the application, which is composed of the loaded dataset, selected molecules, a cached part of the background hierarchy, images of scaffolds and molecules which were already computed, settings of the scaffold components (e.g. sort order, function used to calculate colors in the tree map,...), and some other minor details. Almost all of the application state is stored centrally (using Diode), which is an approach recommended for React. The components themselves are then mostly stateless and straightforwardly transform input properties to resulting DOM.

Another two libraries employed are uPickle (serialization and deserialization) and Autowire (Ajax/RPC), both described in subsection 3.3. The client also uses another serialization library - boopickle - which is by the same author as Diode and almost the same as uPickle but it uses a binary serialization format instead of JSON. In the application boopickle is used to save currently open set of molecules, including the information about scaffolds, significantly saving time on reopening.

Besides that, the client application also uses Bootstrap CSS library, more precisely its Bootstrap-Sass port. Bootstrap is used mostly as a basic CSS stylesheet (which is then heavily customized). Besides the stylesheet, Bootstrap also provides functionality to show modal dialogs, where it is accompanied by jQuery (this is, however, the only place where jQuery is used). A set of icons - Glyphicons - which is included with Bootstrap, is also used. Also the scala-css library is used, provided CSS mixins to React components - in our case mostly just strongly typed access to CSS class names, preventing typos.

Same as in the previous project, code in the client project is also divided into packages:

components In the *components* package, all the React components are contained - i.e. everything that is shown is implemented here. Accompanying the components, contained in subpackage *common*, are helper classes providing common functionality shared by the components. React components are composable and this is heavily used, breaking code apart into smaller pieces. As there are seventeen components in total, we shall not describe them all; instead we describe functionality of one

of the components to give a grasp of what a React component is. An overview of all the components and their relations is show in figure 1.

A nice example of a component is the *ListBox* component, that is used to show the list of molecules and the list of scaffolds (alternative to the tree map). Besides rendering the lists to the DOM (including formatting, event handlers,...), *ListBox* provides the pagination functionality. The list of items to be shown is evaluated lazily - the items are provided as a lazy sequence, and only the items to be shown are fully evaluated. If a single item in the list is changed (for example a scaffold is highlighted), only that item is re-rendered - this functionality is provided by the ListBoxItem child component, which checks whether its properties have changed before re-rendering itself. That altogether means that rendering of the *ListBox* is instantaneous even for an unfiltered list with hundreds of thousands of items contained. An example of a helper class is the *SvgProvider* class, which handles asynchronous loading of SVG representations of molecules or scaffolds from the server - tracking which images need to be loaded, marking those images as pending (not to be requested multiple times in rapid succession), storing the images to the central cache when they are ready[15]. The *SvgProvider* is used for example by the described *ListBox* components, to only request images for the items that are being displayed (and only when they are first being displayed).

services A lot smaller package is the *services* package. It contains two helpers for the remote procedure calls to the server APIs. That means that they take the input parameters for the call to be made, perform the call and return a future of the call result, ensuring that the future is completed once the response is received. One of the helpers is for the calls that use Autowire and uPickle, which is the absolute majority. The only exception is made for sending user input files to the server - as those files can easily be over hundred of megabytes large, we avoid any processing on the client side and send them directly to the server (using the other helper). That is not only faster but in fact the only alternative possible as serializing such file using uPickle would simply crash the browser (and also multiply the size of the data several times).

[15]As the image cache is part of the centralized state of the application handled by the Diode library, only a part of the functionality is implemented in the SvgProvider itself, the rest being implemented in the corresponding Diode handler.

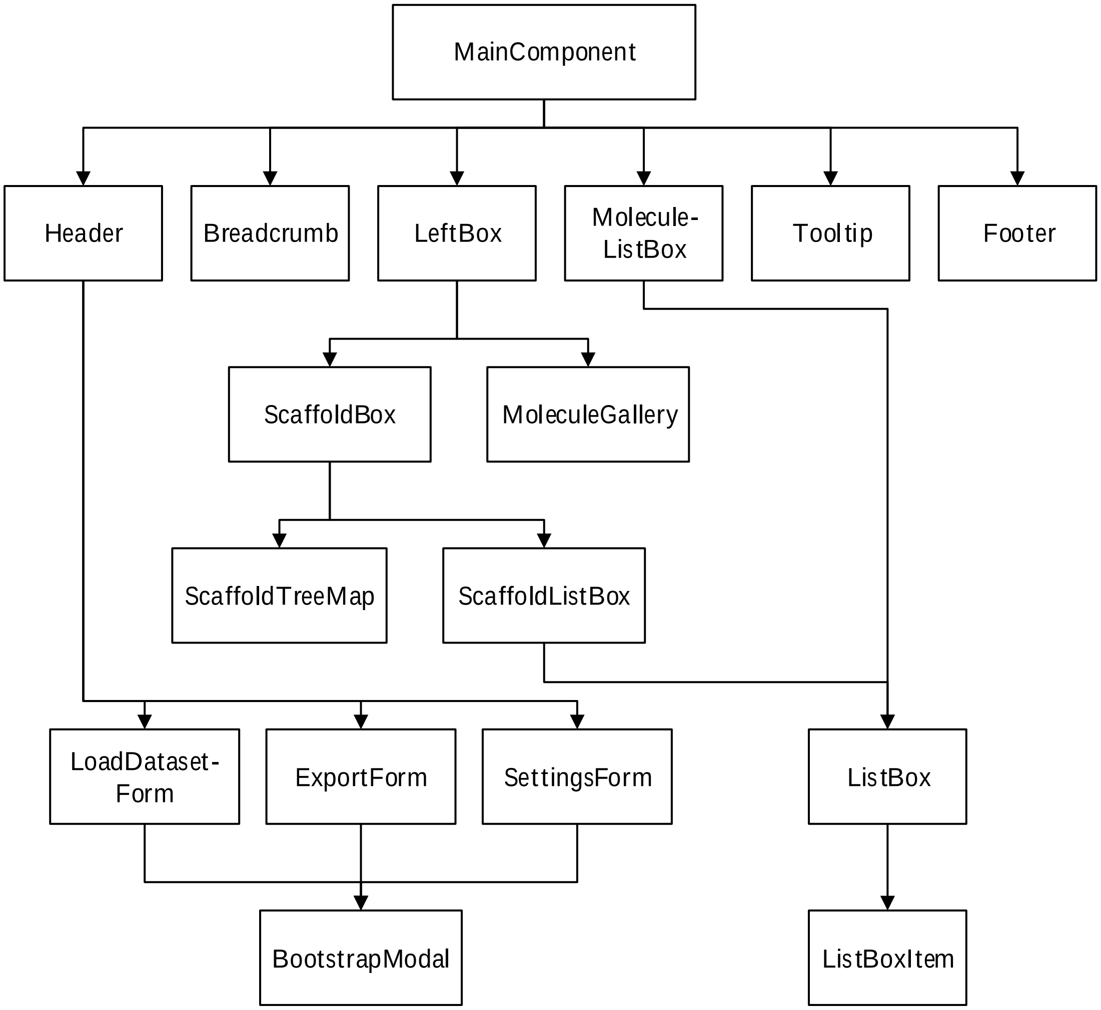

Figure 1: React components in the client project

store Another package of significant size is the *store* package. It contains the *Store* singleton object, which holds the application state. Together with it it contains a *actions*, which specify the changes that are to be performed on the state. Next are the *handlers*, which are the functions that change the state according to the actions. The *handlers* are registered in the *Store* object, which then accepts a stream of *actions* and processes them one by one in a serialized manner. Next is the subpackage *model*, which contains data structures (classes) that describe the central state. Last is the subpackage *serializer* which provides serialization and deserialization of the application state for the purpose of saving to a file or loading from it.

util Last is a small package of non-specific functionality to simplify interactions with the browser. One example might be reading of a file, i.e. converting a JavaScript *File* into a future of byte array, which is used when loading the application state from a file. Another example is a helper to create dataURIs[16] and objectURLs[17], used for displaying images and when saving the application state or exporting the molecules or scaffolds.

3.5 Shared

The last project - *shared* - is a small one. The code it contains is then available in all the three main projects. This is absolutely needed for the interfaces and data structures shared by the client and server side of the application - specifically the interface of the server API (used by Autowire on both ends) and the data structures describing Molecules and Scaffolds (used almost universally).

The rest of the code is generic functionality that can be useful in multiple parts of the application. One example is the Squarified TreeMap [1] layout, which is used by the scaffold map on the client, but was originally used in the generator when prototyping the hierarchy. Another piece of interesting code is the *ReusableComputation* helper, which wraps an ordinary function and adds caching capabilities to it - the last input and result are remembered and if the function is called again with the same parameters, the cached result is returned. That is immensely useful especially in the context of the

[16]https://developer.mozilla.org/en-US/docs/Web/HTTP/Basics_of_HTTP/Data_URIs
[17]https://developer.mozilla.org/en-US/docs/Web/API/URL/createObjectURL

React components in the client application - not only it saves time by not performing the computation again, but it also keeps the result referentially equal to the previous result, which then enables very fast equality checks in subcomponents, which in turn allows to prevent unnecessary re-rendering, ultimately increasing fluency of the application by a noticeable amount.

This concludes our overview of the implementation details. For whoever might be interested in more detailed information, the code itself should be the ultimate reference. We attempted to keep the code concise, structured, following the DRY[18] principle and lacking any surprises (other than where documented). Hopefully, that should make the code comprehensible and even customizable and maintainable. At the same time, the preceding subsections should serve as a practical guide for anybody set to exploring the code for himself.

References

[1] Mark Bruls, Kees Huizing, and Jarke J. van Wijk. Squarified treemaps. In *Eurographics*, pages 33–42. Springer Science+Business Media, 2000. doi: 10.1007/978-3-7091-6783-0_4. URL `https://www.win.tue.nl/~vanwijk/stm.pdf`.

[2] Sébastien Doeraene. Scala.js: Type-Directed Interoperability with Dynamically Typed Languages. Technical report, EPFL, 2013. URL `https://infoscience.epfl.ch/record/190834/files/scalajs-paper.pdf`.

[3] Chris Lattner and Vikram Adve. LLVM: A compilation framework for lifelong program analysis & transformation. In *International Symposium on Code Generation and Optimization, 2004. CGO 2004.*, pages 75–86. Institute of Electrical & Electronics Engineers (IEEE), 2004. doi: 10.1109/cgo.2004.1281665. URL `http://dx.doi.org/10.1109/cgo.2004.1281665`.

[4] Bill Venners Martin Odersky, Lex Spoon. *Programming in Scala*. Artima Inc, 2016. ISBN 0981531687. URL `http://www.ebook.de/de/product/26377173/martin_odersky_lex_spoon_bill_venners_programming_in_scala.html`.

[18] Don't repeat yourself.

[5] Noel M. O'Boyle. cheminformatics.js: Preamble. Online; accessed 2016-07-19, feb 2015. URL `http://baoilleach.blogspot.cz/2015/02/cheminformaticsjs-preamble.html`.

[6] Alon Zakai. Emscripten. In *Proceedings of the ACM international conference companion on Object oriented programming systems languages and applications companion - SPLASH '11*. Association for Computing Machinery (ACM), 2011. doi: 10.1145/2048147.2048224. URL `http://dx.doi.org/10.1145/2048147.2048224`.
