# Supplementary material for: Scaffold analysis of PubChem database as background for hierarchical scaffold-based visualization
Source: J Cheminform. 2016 Dec 29;8:74. doi: 10.1186/s13321-016-0186-7 (PMC5199768; doi:10.1186/s13321-016-0186-7)
Supplement: Supplementary file 2 — Additional file 2. Generating the PubChem background hierarchy with Scaffvis Generator. Description of tasks used for generating the PubChem background hierarchy. [file 13321_2016_186_MOESM2_ESM.pdf]

Generating the Pubchem background hierarchy with Scaffviz Generator

Jakub Velkborsky, David Hoksza

The generator operates with so called *tasks*. The background hierarchy can be obtained by a sequence of three such tasks:

ImportPubChem The first task converts the PubChem Compound database from the form of compressed SDF files, tens of gigabytes large (downloadabe at `ftp://ftp.ncbi.nlm.nih.gov/pubchem/Compound/CURRENT-Full/SDF/`), to a simplified custom database. In this database, each compound is stored as a record consisting of a unique identifier (PubChem Compound ID) and the compound's representation in SMILES format. The database is compressed, 40× smaller than the original form and faster to access. More importantly, it serves as a standardized input format for the following task. This implies that any other database can be used as a source for the background data just by implementing a custom task akin to `ImportPubChem` - i.e. such that it provides a unique identifier and a SMILES string for each molecule in the source database - which should be easy enough.

GenerateScaffolds The second task simply processes the input molecules one by one and calculates their scaffolds. The scaffolds are calculated by level, bottom up. This way, for each unique scaffold the appropriate transformation is performed only once - i.e. if two input compounds share a scaffold on some level, this shared scaffold's parent is computed only once and not once for each of the molecules. The task creates a raw *processing hierarchy*. In this hierarchy, for each scaffold its parent scaffold is stored. As additional information, the number of compounds that corresponds to each scaffold is calculated. All the scaffolds are represented by SMILES strings, unique per level.

GenerateHierarchy The final task converts the processing hierarchy into its final form, which is more compact and suitable to be used by the visualizer. The task consists of two steps. In the first step, each scaffold is assigned a unique numerical id (a primary key) and is stored under this id. In the second step, two maps are created over the ids - a *children* map a *parent* map. The children map contains for each scaffold a list of ids of its children, the parent map contains for each scaffold the unique id of its parent scaffold.

The three tasks can be executed one by one or all at once, as described in the installation guide.

The calculations are performed in parallel, using all available CPU cores. As a result, on a common 4-core 4 GHz processor, using PubChem Compound as the source, the `ImportPubChem` task takes approximately 4 hours and 15 minutes to complete, the `GenerateScaffolds` task takes about 5 hours 35 minutes and the `GenerateHierarchy` task can be completed in about under 25 minutes.
