# Supplementary material for: Scaffold analysis of PubChem database as background for hierarchical scaffold-based visualization
Source: J Cheminform. 2016 Dec 29;8:74. doi: 10.1186/s13321-016-0186-7 (PMC5199768; doi:10.1186/s13321-016-0186-7)
Supplement: Supplementary file 3 — Additional file 3. Scaffvis interface components description. Detail description of interface components. [file 13321_2016_186_MOESM3_ESM.pdf]

Scaffvis interface components description

Jakub Velkborsky, David Hoksza

Description of Scaffvis interface components.

Header The header toolbar at the top of the screen provides buttons opening the dialogues for import or export of data. Next to it are two buttons which switch between a tree map visualization of scaffolds and a scaffold list. Accompanying them is a third button which provides additional settings for the scaffold box. At the right of the header are three buttons enabling and disabling three filters to be applied to molecules in the molecule box. Last is the input box to enter search queries for the molecule box.

Breadcrumb At the left of the screen is the breadcrumb navigation bar which displays the current position in the hierarchy. The bottom most, and slightly darker, scaffold in the breadcrumb is the currently selected scaffold - i.e. the scaffold whose children are shown in the scaffold box next to bread crumb. The unique id of this current scaffold is shown in the browser's address and can be used to revisit it later. The current scaffold is also used to filter molecules in the molecule box are filtered (as long as the "show only subtree" filter is active). Above the current scaffolds are all his ancestors, up to the hierarchy root. Clicking them selects them as the current scaffold. Hovering the mouse above any of the scaffolds shows the tooltip.

Scaffold Box The scaffold box show the children of the current parent scaffold. These children scaffolds can be shown either as the tree map (the default) or the scaffold list. Both can be used to navigate the hierarchy up and down and show information about the scaffolds and the current dataset. But the form is different in both cases, so they will be described separately:

Tree Map The scaffold tree map is the hierarchical visualization that we have been designing throughout this whole work. It displays a set of scaffolds, each scaffold represented by a rectangle. In the rectangle is shown a picture of the scaffold. The rectangle's area is by default based on the relative frequency of the scaffold in the background hierarchy; the color is based on the frequency of the scaffold on the user data set. Sources of the area and color can be changed in the Settings form. Optionally, a logarithmic transformation can be applied. And the color gradient can be replaced by a different one. Clicking a scaffold selects or deselects corresponding molecules. On mouse wheel scrolling the tree map is zoomed in (navigating to the scaffold under the mouse pointer) or zoomed out (navigating to the current scaffold's parent). Moreover, upon hovering the mouse above a scaffold the tooltip is shown providing detailed information.

Scaffold List The functionality of the scaffold list is analogous to that one of the scaffold tree map. The form is, however, different. The scaffolds are shown as a list, sorted lexicographically or by their frequency in the user data set or in the background hierarchy. Each item in the list contains a small image of the scaffold, its name, level in the hierarchy and the number of molecules in the user data set and in the background hierarchy, that correspond to the particular scaffold. A button for "zooming in" the scaffold is present. And clicking the scaffold selects the corresponding molecules, same as in the tree map.

Tooltip An important part of the scaffold tree map is the tooltip. It provides the same information as the scaffold list, together with a larger picture. A picture-only tooltip is also available in the scaffold list and the molecule list.

Molecule List The molecule list is empty until a user data set is loaded. After that, it shows the list of loaded molecules. By default, the list is restricted to the molecules in the current subtree - i.e. the molecules that correspond to the current scaffold. The list can also be restricted to show only the selected molecules or to only show search results - as long as a search query is entered. All the three filters can be toggled using the buttons at the right side of the header. The search query can be entered in the same place. For each molecule a picture is shown, together with its SMILES string and its name and comment, as long

as they were present in the input. Clicking a molecule selects it. The molecules are shown by pages consisting of 50 items. The total number of pages is not precalculated to avoid evaluation of the filters on huge data sets - i.e. only the next page and the preceding pages are available in the pager.

Footer The footer shows the size of the loaded data set and the size of the current subtree. If any molecules are selected, it also shows the number of selected molecules in both - the data set and the current subtree.

Export Form The Export form is accessible via the "Export" button in the toolbar. It allows exporting molecules or scaffolds in standard SMILES format or exporting the whole dataset in a custom binary format.

Load Dataset Form The Load Dataset form is accessible clicking the "Open" button in the toolbar. It allows selecting a file containing user molecules to be loaded; the file is then submitted to the server. The file can be in arbitrary format which is supported by the ChemAxon JChem cheminformatics toolkit, or it can be the binary dataset obtained through the Export form, which is considerably faster to load (as the scaffolds are already precomputed).

Settings Form The Settings form, also accessible via the the header toolbar, offers a range of settings for the Scaffold Box.
